# Genome-wide expression analysis of *carboxylesterase* (*CXE*) gene family implies *GBCXE49* functional responding to alkaline stress in cotton

**DOI:** 10.1186/s12870-022-03579-9

**Published:** 2022-04-12

**Authors:** Cun Rui, Fanjia Peng, Yapeng Fan, Yuexin Zhang, Zhigang Zhang, Nan Xu, Hong Zhang, Jing Wang, Shengmei Li, Tao Yang, Waqar Afzal Malik, Xuke Lu, Xiugui Chen, Delong Wang, Chao Chen, Wenwei Gao, Wuwei Ye

**Affiliations:** 1 Institute of Cotton Research of Chinese Academy of Agricultural Sciences / Research Base, State Key Laboratory of Cotton Biology, School of Agricultural Sciences, Zhengzhou University, Henan 455000 Anyang, China; 2 Hunan Institute of Cotton Science, 3036 Shanjuan Road, Changde, 415101 China; 3 Engineering Research Centre of Cotton, Ministry of Education / College of Agriculture, Xinjiang Agricultural University, 311 Nongda East Road, 830052 Urumqi, China

**Keywords:** Carboxylesterase (CXE), *Gossypium barbadense*, Collinearity, Abiotic stress, Differential expression

## Abstract

**Background:**

Carboxylesterase (CXE) is a type of hydrolase with α/β sheet hydrolase activity widely found in animals, plants and microorganisms, which plays an important role in plant growth, development and resistance to stress.

**Results:**

A total of 72, 74, 39, 38 *CXE* genes were identified in *Gossypium barbadense*, *Gossypium hirsutum*, *Gossypium raimondii* and *Gossypium arboreum*, respectively. The gene structure and expression pattern were analyzed. The *GBCXE* genes were divided into 6 subgroups, and the chromosome distribution of members of the family were mapped. Analysis of promoter cis-acting elements showed that most *GBCXE* genes contain cis-elements related to plant hormones (GA, IAA) or abiotic stress. These 6 genes we screened out were expressed in the root, stem and leaf tissues. Combined with the heat map, *GBCXE49* gene was selected for subcellular locate and confirmed that the protein was expressed in the cytoplasm.

**Conclusions:**

The collinearity analysis of the *CXE* genes of the four cotton species in this family indicated that tandem replication played an indispensable role in the evolution of the *CXE* gene family. The expression patterns of *GBCXE* gene under different stress treatments indicated that *GBCXE* gene may significantly participate in the response to salt and alkaline stress through different mechanisms. Through the virus-induced gene silencing technology (VIGS), it was speculated that *GBCXE49* gene was involved in the response to alkaline stress in *G. barbadense*.

**Supplementary Information:**

The online version contains supplementary material available at 10.1186/s12870-022-03579-9.

## Background

Thousands of metabolites were synthesized in higher plants, these metabolites played an important role in plant growth and development, hormone signal transmission, and stress defense response [1]. Plant hormones are more important in the process of regulating growth, development and coping with external environmental stress. Many studies have focused on enzymes that catalyze acylation to esters. However, in ester metabolism, the reverse reaction of hydrolyzing ester bonds is also common. The enzyme responsible for this process was called carboxylesterase (CXE, EC 3.1.1.1). Carboxylesterase (CXE) is a class of hydrolytic enzymes that are widely found in animals, plants and microorganisms and can catalyze esters and amide compounds. At present, 20, 16, 6, 33 *CXE* genes have been found in *Arabidopsis thaliana* [2], *Malus domestica* [3], *Vitis flexuosa Thunb* [4], *Prunus persica* [5] and other plants, respectively, these *CXE* genes were actively involved in different developmental processes. CXE had a variety of physiological functions, such as participating in the degradation of nerve signal transmission pheromones and the degradation of other harmful foreign bodies in animals [6]. In plants, CXE protein plays an important role in herbicide detoxification [7], activation of hormone signal substances [8], and response to biological stress.

If a type of protein or gene is a domain with a common origin, it belongs to a gene family, superfamily refers to the same gene can belong to two or more gene families [9]. Plant CXE proteins were a supergene family [10], and played an important role in the regulation of herbicide activity, the activation of plant hormone signal substances, and the response of plants to biological stress [11]. The expression of plant carboxylesterase genes at the transcriptional level was very complex, with both constitutive expression and tissue-specific expression as well as inducible expression of bacterial hormones. Hormone signal molecules in plants usually regulate the growth and development of plants through the conversion between inactive esters and active molecules. When plants need, they will be selectively hydrolyzed by esterases to release these signal molecules [12]. Structurally, CXE belonged to the α/β sheet hydrolase superfamily, the family members contained a conserved core structure consisting of 8 β sheet α helices and loop structure [2]. Researchers isolated two carboxylesterase (*AtCXE*) genes, *AtCXE8* and *AtCXE9* from *Arabidopsis thaliana*, the AtCXE8 and AtCXE9 proteins possess carboxylesterase motifs (-GXSXG-) and catalytic triads (Ser, Asp, and His). Overexpression of the *AtCXE8* gene in transgenic *Arabidopsis* plants led to enhanced disease resistance against B. cinerea [13]. Similarly, in the fruits of pepper, it was found through immunochemical experiments that *PepEST* genes containing -HGGGF-and-GXSXG-motifs and a catalytic triad, when the fruit is infected with *C. gloeosporioides*, *PepEST* accumulation was localized in epidermal and cortical cell layers in infected ripe fruit, but rarely even in epidermal cells in infected unripe one, so it proved that *PepEST* was involved in the resistance of ripe fruit against *C. gloeosporioides* infection [14].

Early studies found that plant CXE isoenzymes were widely present in different tissues, organs, developmental stages and different parts of cells [15]. Carboxyesterase has been studied in many physiological processes of plants. Twenty carboxylesterase genes were identified from the model plant *Arabidopsis*, which are widely present in the *Arabidopsis* genome (4 of 5 chromosomes), 18 of which were expressed in most tissues of *Arabidopsis* plants, *AtCXE13* was only expressed in flowers and fruits, *AtCXE1* was not expressed in leaves at all stages [2]. The carboxylesterase gene cloned from licorice participated in the oxidation reaction of hydroxyisoflavone dehydrogenase [16]. Among the 16 *CXE* genes identified from *Malus domestica*, *MdCXE1* and *MdCXE12* had very high homology with *Arabidopsis AtCXE18* genes, while *MdCXE12* was more closely related to rice gibberellin receptor GID1. *MdCXE1* was in the early stage of *Malus* domestica fruit development, it was expressed at a low level of harvested and mature, while a highest level in the fruit, it may affect the flavor of *Malus domestica* through the ability to hydrolyze the relevant flavor esters in the mature fruit. During the entire fruit development process, *MdCXE16* was in the wild constitutive expression [17]. In addition, the GA receptor GID1 was also a carboxylesterase protein, whose function was to induce degradation of ubiquitin and GA transcription inhibitors by binding to GA [18]. The enzyme in *Actinidia eriantha* (*AeCXE1*) can hydrolyze a range of carboxylester substrates with acyl groups ranging from C2 to C16, with a preference for butyryl moieties [19]. This diversity of expression indicated that plant carboxylesterase genes played an important role in the process of plant growth and development and biological stress.

*Gossypium barbadense*, as a cultivated species of *Gossypium*, is well-known not only for high-quality fibers, but also for pioneer species in the saline and alkaline land. However, the growth and yield of cotton will be severely restricted by external and internal environmental factors. More and more studies have shown that CXE family proteins play an important role in hormone signal transduction, defense response and participation in biological stress. There are few studies on the CXE protein in the process of cotton growth and development. This study conducted a comprehensive review of the cotton CXE family, and initially identified the *CXE* genes of the four major cotton species. In *G. barbadense*, one of the *CXE* genes was identified. Further research was carried out, and a preliminary identification of the alkali resistance of the gene was made. This study provided a new perspective for the evolution and in-depth study of this gene family by analyzing the possible involvement of some *CXE* genes in environmental stress tolerance. Our results provide an identifiable basis for further study of the mechanism of action of this family of genes and the biological function of *CXE* gene in the growth and development of *G. barbadense*.

## Results

### Identification of *CXE* family members

To identify the members of the *CXEs* family in cotton, we used the hidden Markov model of PF07859 as the query condition, and compared the proteome and genome of *G. barbadense*, *G. hirsutum*, *G. arboreum*, and *G. raimondii* by using local blast software. Screening was performed according to the structural domain Abhydrolase_3, and the genes that are incomplete and do not contain this structural domain are eliminated to determine the family members. We finally identified 72, 74, 38, 39 genes in *G. barbadense* (*GBCXEs*), *G. hirsutum* (*GHCXEs*), *G. arboretum* (*GaCXEs*), and *G. raimondii* (*GrCXEs*), respectively (Fig. S1). Based on the number of genes in the four cotton species, it was determined that the cotton *CXE* gene was a conservative type, which was basically consistent with the evolution of allotetraploid cotton [20], they were named *GBCXE1-72*, *GHCXE1-74*, *GaCXE1-38*, *GrCXE1-39* according to their position on the chromosome.

The physical and chemical properties of the *CXE* family members (protein length, protein molecular weight (MWs), isoelectric point (pIs), and protein hydrophilicity and hydrophobicity) were analyzed (Table S1). The results showed that in *G. barbadense*, the length of the protein encoded by this family member ranged from 261 aa (*GBCXE25*) to 462 aa (*GBCXE71*), MWs ranged from 28.44 kDa (*GBCXE25*) to 50.86 kDa (*GBCXE34*), and pIs ranged from 4.85 (*GBCXE7*) to 9.15 (*GBCXE68*), the range of the charge was -10.5–15, and the hydrophilic and hydrophobic coefficients were all negative, which proved that the proteins in this family were all hydrophilic proteins. Among the three family members of *GHCXEs*, *GaCXEs*, and *GrCXEs* of other cotton species, the physicochemical properties of the encoded proteins ranged from 227 aa (*GHCXE72*) to 462 aa (*GHCXE73*), 260 aa (*GaCXE6*) to 462 aa (*GaCXE35*), 244 aa (*GrCXE9*) -462 aa (*GrCXE38*), MWs from 25.77 kDa (*GHCXE72*) -50.86 kDa (*GHCXE34*), 29.33 kDa (*GaCXE6*) -50.77 kDa (*GaCXE35*), 27.10 kDa (*GrCXE9*)-50.75 kDa (*GrCXE38*). PIs ranged from 4.85 (*GHCXE7*)-9.48 (*GHCXE70*), 4.91 (*GaCXE8*)-9.10 (*GaCXE31*), 4.99 (*GrCXE32*)-9.36 (*GrCXE27*), the charge number ranged from -10.5 to 16.5, -9.5–15, 8.5–16, all family members were hydrophilic proteins. The prediction of subcellular localization shown that there were 46 genes in the *GBCXEs* family were located at the cytoplasm, 2 at the extracellular matrix, 4 at the inner membrane, 2 at the outer membrane, and 18 at the periplasmic.

### Analysis of evolutionary relationship of family members

**Fig. 1:**
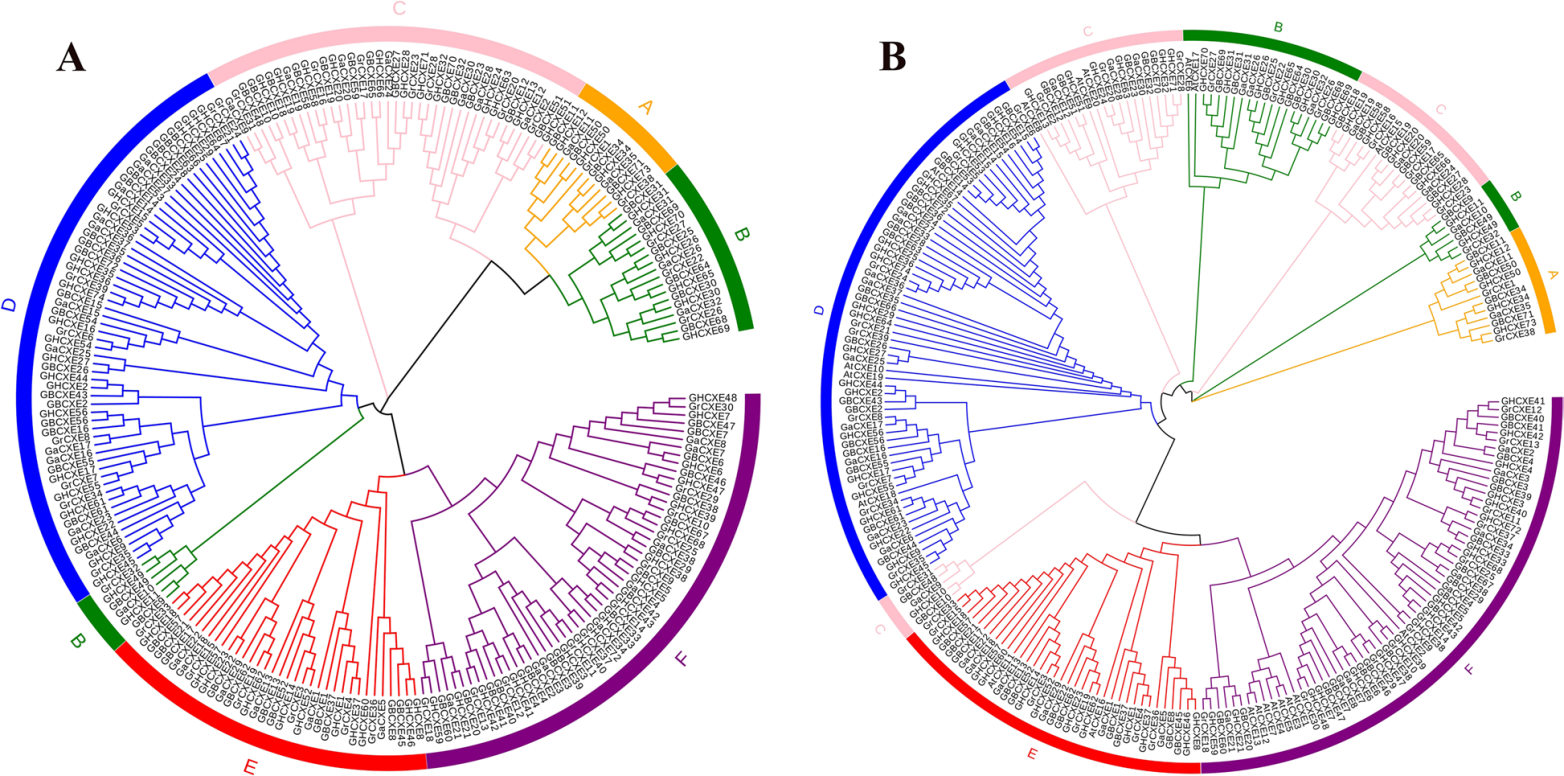
Phylogenetic tree of *CXE* family members. **A** Four cotton species (*G. barbadense*, *G. hirsutum*, *G. arboreum*, *G. raimondii*) *CXE* gene phylogenetic tree; **B** Four cotton species and *Arabidopsis CXE* gene phylogenetic tree

To study the evolutionary relationship between CXE proteins, 243 CXE protein sequences (Fig. S2) from four cotton species and *Arabidopsis* were used for sequence alignment. By using MEGA7 software, the evolutionary relationship between each sequence was constructed and the rootless phylogenetic tree was generated (Fig. 1B), the CXE protein was divided into 6 subgroups according to the number and structural characteristics of the domains in each sequence. Among them, the D and F subgroups contain a larger number of genes, with 19 and 17 *GBCXEs* in them, respectively. After carefully checking the 6 subgroups, it was not difficult to find that in *Arabidopsis*, except for the A subgroup did not contain the *AtCXEs* gene, the other six subgroups had the *Arabidopsis* *CXE* gene.

In addition, to study the relationship between the common ancestors of diploid and allotetraploid cotton, we have constructed four phylogenetic trees of CXE proteins of cotton species (Fig. 1A). Referring to the subgroups, we found that the *CXE* genes of the four cotton species were distributed in each subgroup, and each branch contains proteins from diploid and allotetraploid cotton species. Based on the number of genes contained in the four cotton species in the 6 subgroups, we found that the number of tetraploid species was almost twice that of diploid species. These clustering data showed that the tetraploid species *G. hirsutum* was twice as many as two species. The results of hybridization of cotton species (*G. arboreum*, *G. raimondii*) provide evidence. Interestingly, the homologous genes of *G. barbadense*, whether it was *G. arboreum* or *G. raimondii*, a large proportion of the CXE protein was carried out under the influence of stable selection, so these genes evolve slowly after repeated events, and conservation at the protein level. In the evolutionary tree, all species have gene pairs from the same node, which indicates that the *CXE* genes of all the species we studied have experienced gene duplication events, which eventually led to the expansion of *CXE* genes. However, in different species, the gene duplication between different groups was different. The genes number of *G. hirsutum* and *G. barbadense* was much higher than that of other species, indicating that the *CXE* gene family in these two cotton species has shown a large-scale expansion during the evolution process. These results indicated that gene duplication was the main reason for the expansion of the *CXE* gene family in plants.

### Distribution of *CXE* gene family members on chromosomes

**Fig. 2:**
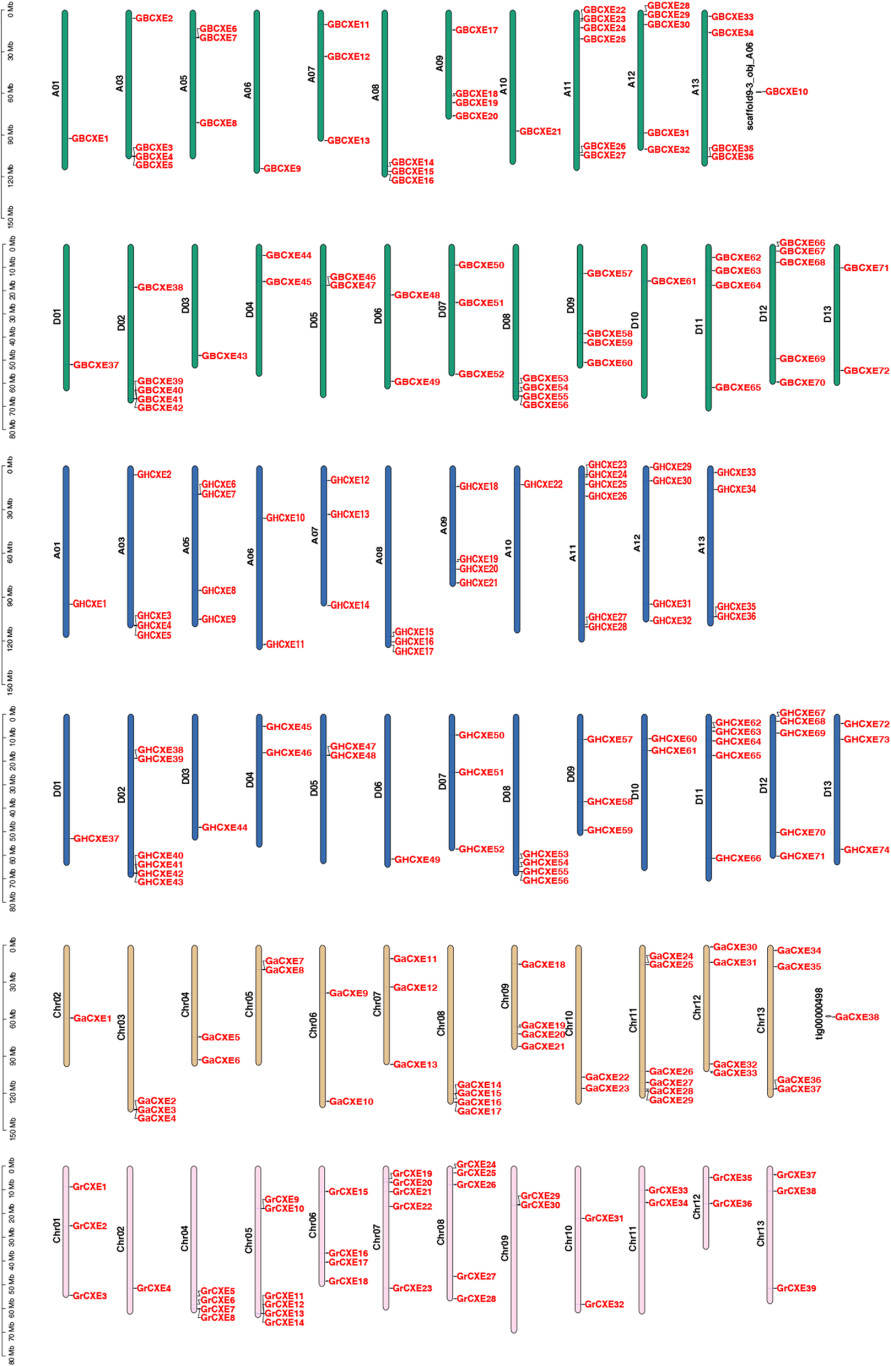
Chromosomal location of *CXE* genes in four cotton species

The gff3 file of the genome and the gene ID information were used to drew the chromosome distribution map of the family members through the TBtool software (Fig. 2). The *CXE* genes of *G. barbadense*, *G. hirsutum*, *G. arboreum*, and *G. raimondii* were located on the chromosomes of the corresponding cotton species, 221 family members were located on different specific chromosomes, of which only *GBCXE10*, *GaCXE38* gene were mapped to scaffold. Through the distribution of genes and subgenome of allotetraploid species [21, 22], we found that one gene was located at a similar position on Chr01 of subgroup A and subgroup D of *G. barbadense*, this discovery was the same as *G. hirsutum*, and in terms of evolutionary relationship, *GBCXE1* and *GBCXE37* gene were a homologous gene, and the two genes had similar promoter elements (Fig. 6). The number of genes distributed in subgroup A and subgroup D of this cotton species was the same, and the number of genes distributed on chromosomes A11, A12, D02, D08 and D12 was more than other chromosomes.

In *G. hirsutum*, the number of genes on the chromosomes of the two subgroups was the same, the number of genes distributed on chromosomes 3, 5, 9, 11, 12, 13 of subgroup A and chromosomes 2, 8, 11, and 12 of subgroup D was more than other chromosomes. It was noteworthy that there was no gene distribution in the second and fourth chromosomes of the A subgroup of the two tetraploid cotton species. In *G. arboreum*, gene deletion on Chr01, one of the genes was mapped to the scaffold, and the number of genes on Chr08, Chr09, Chr11, Chr12, and Chr13 are 4, 4, 6, 4, 4, respectively. In *G. raimondii*, there were more genes on Chr04, Chr05, Chr06, Chr07, and Chr08, with 4, 6, 4, 5, and 5 genes on the corresponding chromosomes respectively, while Chr02 of this cotton species had no *CXE* family members. The distribution of *CXE* gene on 13 chromosomes of different cotton species was uneven, and the number of genes distributed on each chromosome had no obvious correlation with the length of the chromosome.

### The collinearity of *CXE* gene in cotton

**Fig. 3:**
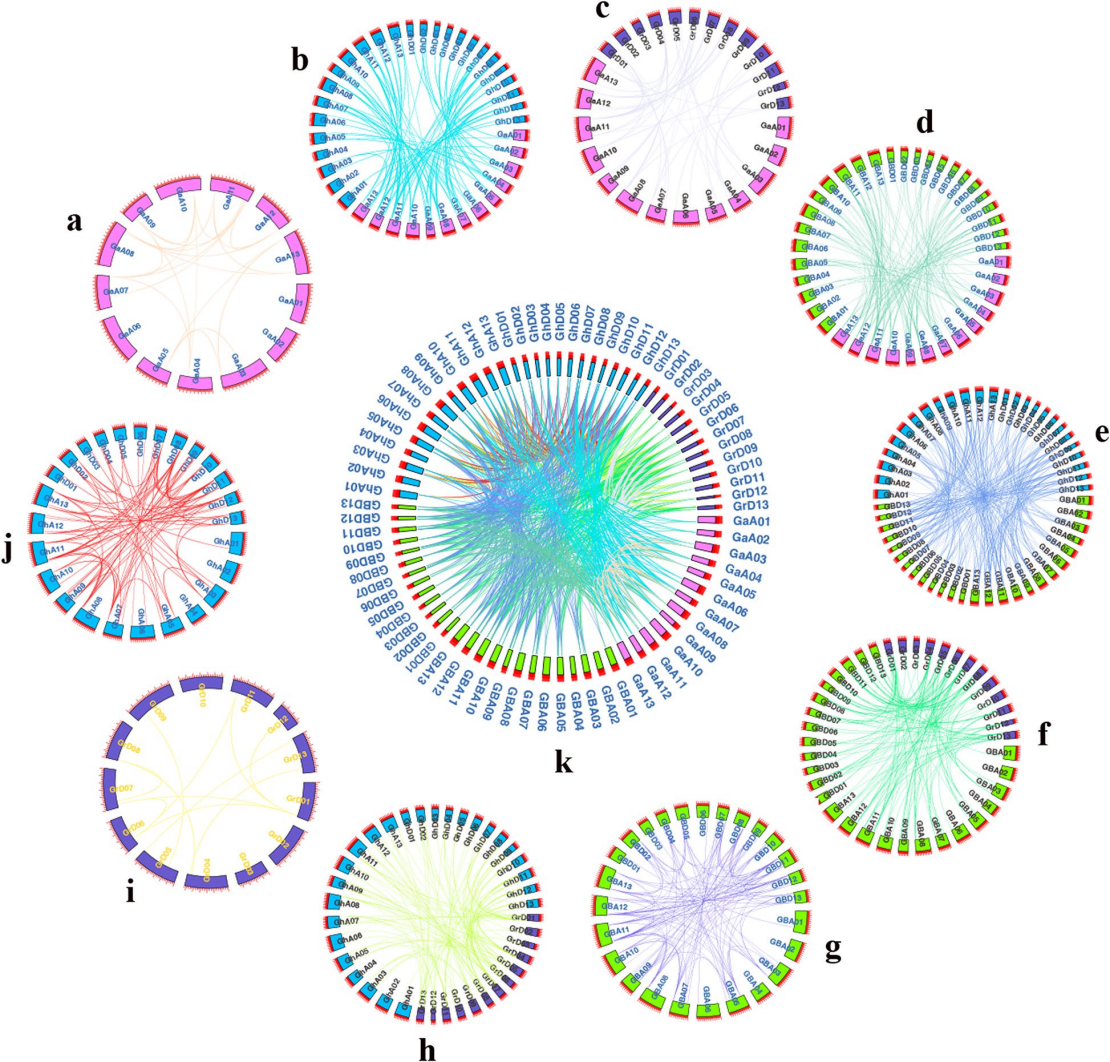
The collinearity of *CXE* genes within and among the four cotton species genomes. Chromosomes and gene pairs were represented by different colors. (**a**: Ga-Ga, **b**: Ga-GH, **c**: Ga-Gr, **d**: Ga-GB, **e**: GB-GH, **f**: GB-Gr, **g**: GB-GB, **h**: Gr-GH, **i**: Gr-Gr, **j**: GH-GH, **k**: summary of 10 combinations of collinearity)

To describe the positional relationship of genes on the same chromosome, the homology of genes, the amplification mechanism and the sequence of arrangement, we performed a collinearity analysis on the *CXE* genes of the four cotton species. The genomic protein sequences of each cotton species were compared, then MCScanX [23] was used to find homologous gene pairs, combined the length files of chromosomes between genomes, and presented with CirCos [24], the result was presented as a circle graph (Fig. 3). A total of 1154 gene pairs (Table S4) in the four cotton species were identified as repeated gene pairs, of which 22 pairs of tandem repeats and 236 pairs of fragment repeats. We found that there are 131 gene pairs in the 72 *CXE* genes of *G. barbadense* (Fig. 3-g), of which 124 pairs are fragment repeats and 7 pairs of tandem repeats (Table S3) (*GBCXE3/4*, *GBCXE35/36*, *GBCXE6/7*, *GBCXE39/40*, *GBCXE40/41*, *GBCXE46/47*, *GBCXE55/56*). Among the 74 *CXE* genes of *G. hirsutum*, there are 90 gene pairs (Fig. 3-j), of which 83 pairs are fragment repeats and 7 pairs of tandem repeats (*GHCXE3/4*, *GHCXE6/7*, *GHCXE35/36*, *GHCXE40/41*), *GHCXE41/42*, *GHCXE47/48*, *GHCXE55/56*). The tandem duplications of the *CXE* genes of the two tetraploid cotton species all occured on chromosomes 3, 5, 13 of subgroup A, and chromosomes 2, 5, and 8 of subgroup D, respectively. Moreover, there were two pairs of tandem repeats on chromosome 2 in subgroup D.

The 40 genes of *G. raimondii* form 12 CXE homologous gene pairs (Fig. 3-i), of which 7 pairs are fragment repeats and 5 pairs are tandem repeats (*GrCXE7/8*, *GrCXE9/10*, *GrCXE11/12*, *GrCXE12/13*, *GrCXE29/30*) occured on chromosomes 4, 5, and 9 of subgroup D, and a total of 3 pairs of tandem duplications occur on chromosome 5. Meanwhile, 39 genes of *G. arboreum* formed 25 *CXE* homologous gene pairs (Fig. 3-a), of which 22 pairs were fragment repeats and 3 pairs of tandem repeats (*GaCXE36/37*, *GaCXE16/17*, *GaCXE2/3*), tandem duplication occured on chromosomes 3, 8, and 13 of subgroup A. These results indicated that fragment duplication is the main cause of gene amplification, and tandem duplication played an indispensable role in the evolution of the *CXE* gene family. Analysis between diploid and diploid (Fig. 3-c), we found that there were 53 gene pairs between *G. arboreum* and *G. raimondii*. For tetraploid, there were 286 gene pairs between *G. barbadense* and *G. hirsutum* (Fig. 3-e), There were 154 gene pairs between *G. arboreum* and *G. barbadense* (Fig. 3-d), and 102 gene pairs with *G. hirsutum* (Fig. 3-b). Simultaneously, there were 159 (Fig. 3-f) and 142 (Fig. 3-h) gene pairs between *G. raimondii* and *G. barbadense*, *G. hirsutum*, respectively.

### Selective pressure Ka/Ks measurement and analysis

**Fig. 4:**
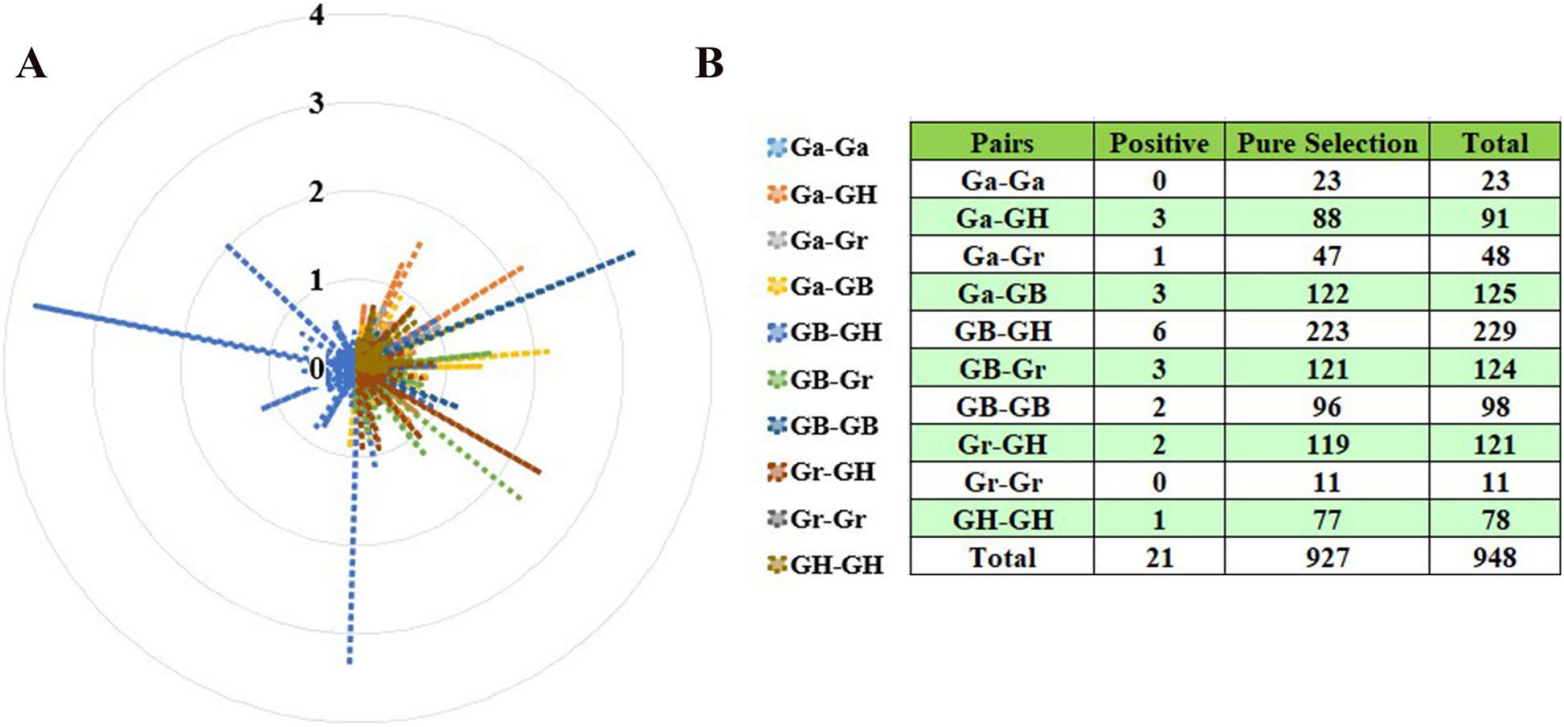
Select the radar chart for pressure (Ka/Ks) analysis. **A** Radar chart of selection pressure. **B** Prediction of no of duplicated gene pairs involved in different combinations from four *Gossypium* species

Non-functionalization (loss of original functions), sub-functionalization (division of original functions), and new functionalization (acquisition of new functions) were the three evolutionary directions of proteins in which replication genes experience functional differences [25]. Calculation of non-synonymous (Ka) to synonymous (Ks) substitution rates was used to infer the size of the selection constraint, and then further study the selection pressure of gene duplication gene pairs in the evolution process. We measured the Ka and Ks and Ka/Ks homologous gene pairs in 10 combinations (Ga-Ga, Ga-GH, Ga-Gr, Ga-GB, GB-GH, GB-Gr, GB-GB, Gr-GH, Gr-Gr, GH-GH) of four cotton species (Fig. 4). Ka /Ks < 1 was considered to be a purification selection, indicating that natural selection eliminates harmful mutations and keeps the protein unchanged, Ka /Ks > 1 was positive selection, indicating that natural selection acts on the change of protein to quickly fix the mutation site in the population and accelerate the evolution of genes, Ka /Ks = 1 was neutral selection, indicating that natural selection has no effect on mutation. [26]. Therefore, we infered the selection pressure of repeated gene pairs based on the ratio of Ka/Ks. The results (Fig. 4) showed that Ka /Ks > 1 for 21 gene pairs (Table S5), among which Ka /Ks < 1 for 927 gene pairs; and 0.5 < Ka /Ks < 0.99 for 78 gene pairs. In our results, more than 90% of gene pairs the *CXE* genes of the four cotton species had a Ka/Ks ratio of less than 1, indicating that the family genes were subject to strong purification selection and high conservation.

### Analysis of G. *barbadense* CXE conservative protein motif and gene structure

**Fig. 5:**
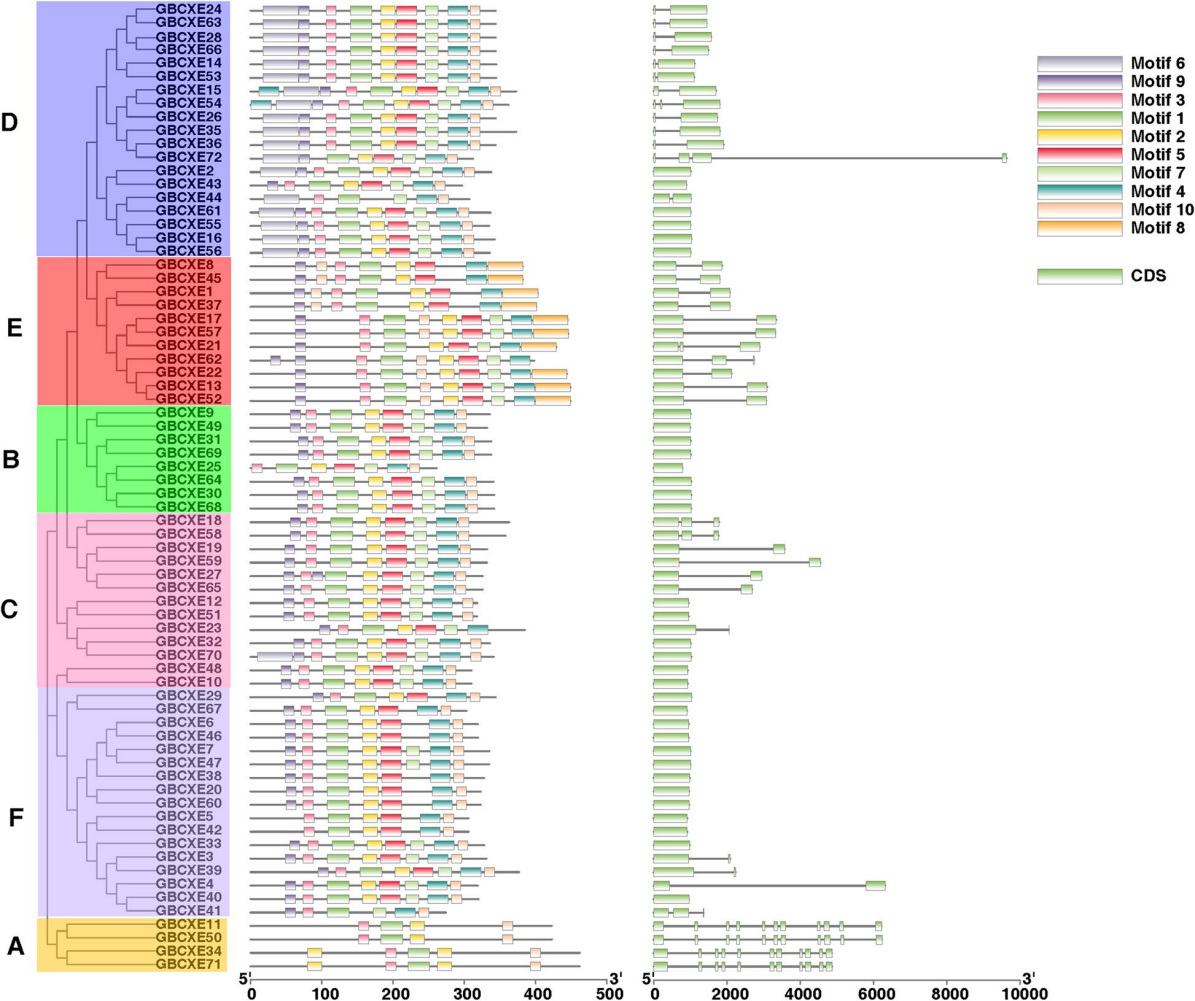
Analysis of phylogenetic tree, conserved domain and gene structure of *GBCXEs*

The motifs of the *G. barbadense CXE* gene sequence were analyzed through the online website MEME. A total of 10 motifs were identified (Fig. 5), these motifs performed different functions and were distributed in the sequence of each subgroup. The gene sequences in subgroup A were relatively simple, containing 4–5 motifs and frequently interrupted introns. The genes in subgroup B basically included the 9, 3, 7, 2, 5, 1, and 10 motifs, and contains only one exon. The C subgroup and the B subgroup had similar motifs, what was interesting was that the *GBCXE70* gene in the C subgroup contained a 6 motif. Members of subgroup D, except for *GBCXE43*, *GBCXE15*, *GBCXE54*, other members except motif 8, other motifs are included in each subgroup. Most members of the E subgroup contained motif 9, 3, 2, 5, 7, 4, and 8, the same F subgroup members mostly contained the 9, 3, 1, 2, 5, 4, 10 motifs, and most members of this subgroup had no introns. There were similar motif arrangements in the same subgroup, revealing that the protein structure was conserved in a specific subgroup, and the functions of most conserved motifs remain to be elucidated. There was little difference in the length of the exons of the sequence encoded by the gene, which indicated that the gene had a certain degree of conservation. Exons/introns were randomly distributed in *GBCXEs* of different evolutionary branches, which also indicated that similar genes were clustered together in the same phylogenetic tree.

### Analysis of promoter Cis-acting elements and expression

**Fig. 6:**
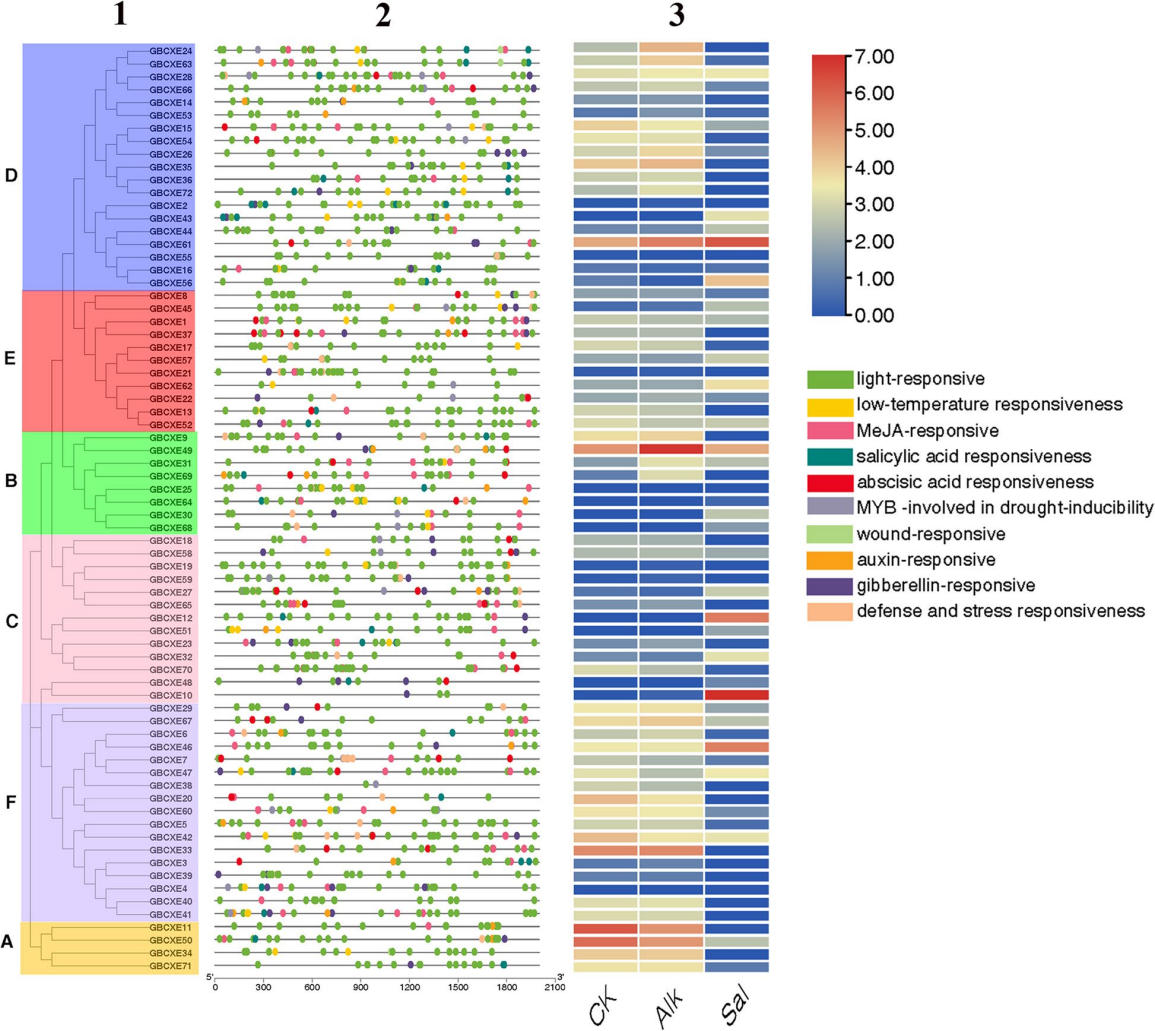
Analysis of promoters and differentially expressed genes of *GBCXEs* family. **1** Phylogenetic tree of *GBCXEs*; **2** Cis-elements in promoters of *GBCXE* genes; **3** Differentially expressed genes of *GBCXE* genes under salt and alkaline stress

In view of the importance of various promoter elements in abiotic stress, by using the evolutionary relationship file of the *GBCXEs* and the 2 kb region file upstream of the start codon of the *GBCXEs*, we identified the plant hormones, cold, heat, light, abiotic stress response element of *GBCXEs* (Fig. 6-2). About half *GBCXE* members were involved in MeJA regulation, most of the members had gibberellin responsive elements, and in subgroup C except for *GBCXE32*, *GBCXE70*, *GBCXE19* other members had gibberellin responsive elements, through an overview of all *GBCXEs* members, all members contained light-responsive elements (Table S6). We found that the number of gibberellin response elements involved in different branches of evolution (Fig. 6-1) was also different. In our results, some *GBCXE* members also contained MeJA response elements, studies have shown that plant CXE protein can demethylate inactive methyl jasmonate to produce active jasmonic acid [27]. The *CXE* gene cloned by the researchers in *Vitis flexuosa* had a protein with methyl jasmonate esterase activity, and under high concentration of methyl salicylate, it also has methyl salicylate esterase activity [28]. We found that the cis-acting elements contained in gene promoters within the same gene subgroup are not the same, even the cis-acting elements contained in homologous genes with high similarity were also different. Through promoter analysis, we can summarize the mechanism of genes responding to different plant hormones, which would help to further understand the regulatory network of the *GBCXE* gene family.

RNA-seq data was used to determine the differences in gene expression of family members under salt and alkaline stress to further explore the response mechanism of *GBCXEs* under abiotic stress (Fig. 6-3) (Table S7, S8), through the results, we can clarify that in addition to the genes *GBCXE4*, *GBCXE19*, *GBCXE59*, *GBCXE25*, *GBCXE64*, *GBCXE21*, *GBCXE55*, *GBCXE2*, other family members have responded to salt and alkaline stress to a certain extent. The expression levels of *GBCXE49*, and *GBCXE31* were up-regulated after stress, and genes in subgroup A and some genes in subgroup D and F were down-regulated after stress. Interestingly, we found that genes such as *GBCXE43*, *GBCXE56*, and *GBCXE62* were particularly responsive to salt stress, however, genes such as *GBCXE49*, *GBCXE24*, *GBCXE69*, and *GBCXE11* respond to alkaline stress, *GBCXE49* had the highest differential expression under alkaline stress. Gene expression changed under different stresses, even though the elements were the same, the functions of gene members were also diverse. These results indicated that *GBCXE* members were involved in the regulation of salt and alkaline stress, the response expression of different members was different, and the expression of the same gene under different stress was also different. We considered that it may be due to the different action elements, the time response mechanism of stress treatment was different.

### Interaction network of GBCXE proteins

**Fig. 7:**
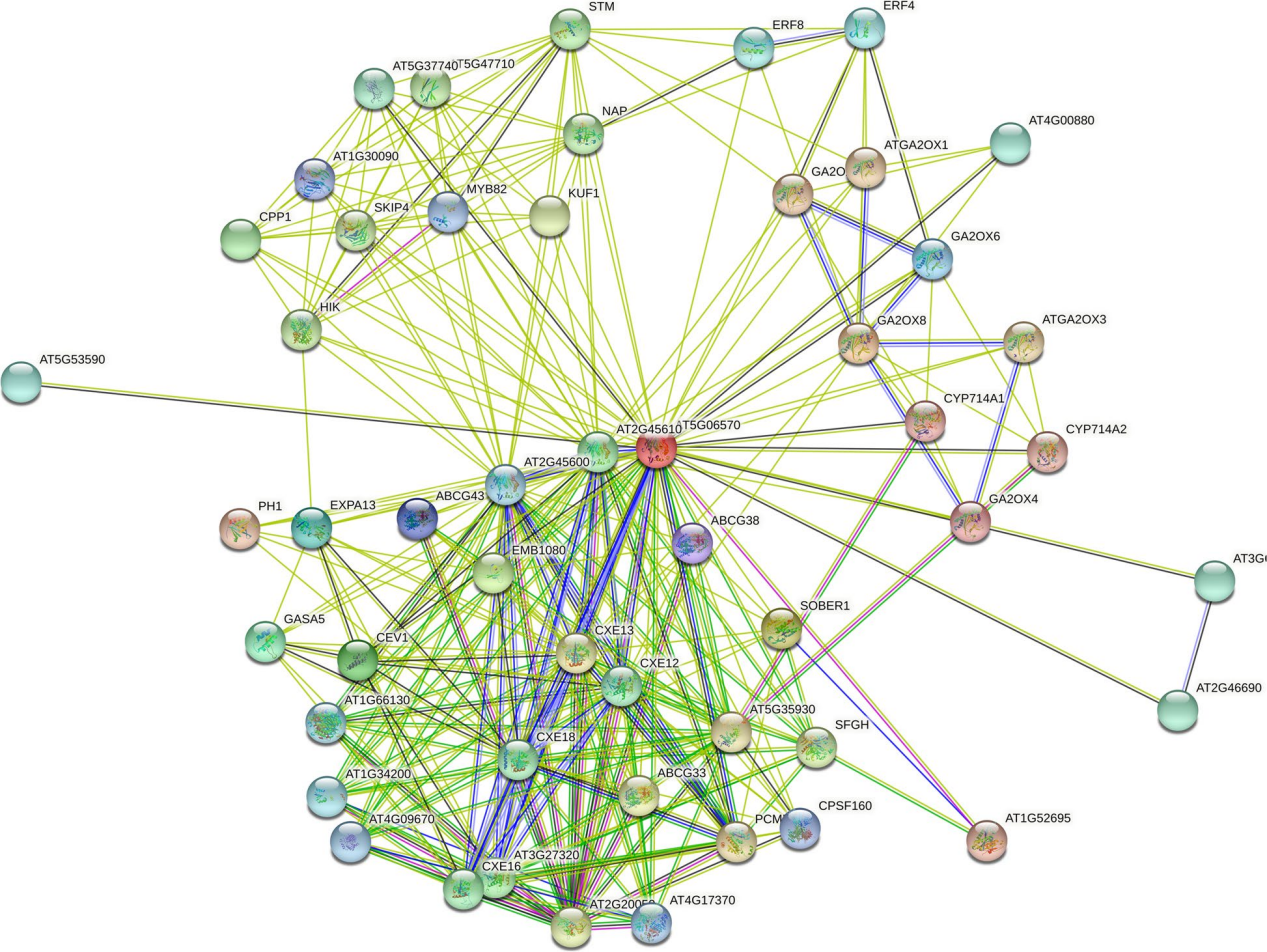
Interaction network of GBCXE proteins

To study the function of GBCXE protein, *Arabidopsis* orthologous genes were used as the basis, protein sequence search was used to performe interaction network analysis (Fig. 7) by using STRING data (https://string-db.org/). The function of GBCXE protein was speculated to be based on the study of AtCXE protein. In our results, the promoters of most *GBCXE* members had GA response elements, and these proteins interact with other pathways. The GBCXE49 homologous AtCXE15 protein (AT5G06570) interacted with CXE13, CXE12, CXE18, and CXE16, also interacted with the proteins GA2ox1, GA2ox2, GA2ox3, GA2ox4, GA2ox6, and GA2ox8. In addition, in the interaction network, the carboxylesterase protein AtCXE15 was also closely related to the ABC transporter, the nucleic acid-binding protein, and the EXPA13 protein that regulated the relaxation and extension of plant cell walls. Combined with our analysis of the CXE protein promoter's action elements and protein network interactions, it would help us understand the protein's mechanism of action.

### Specific expression of *CXE* genes

**Fig. 8:**
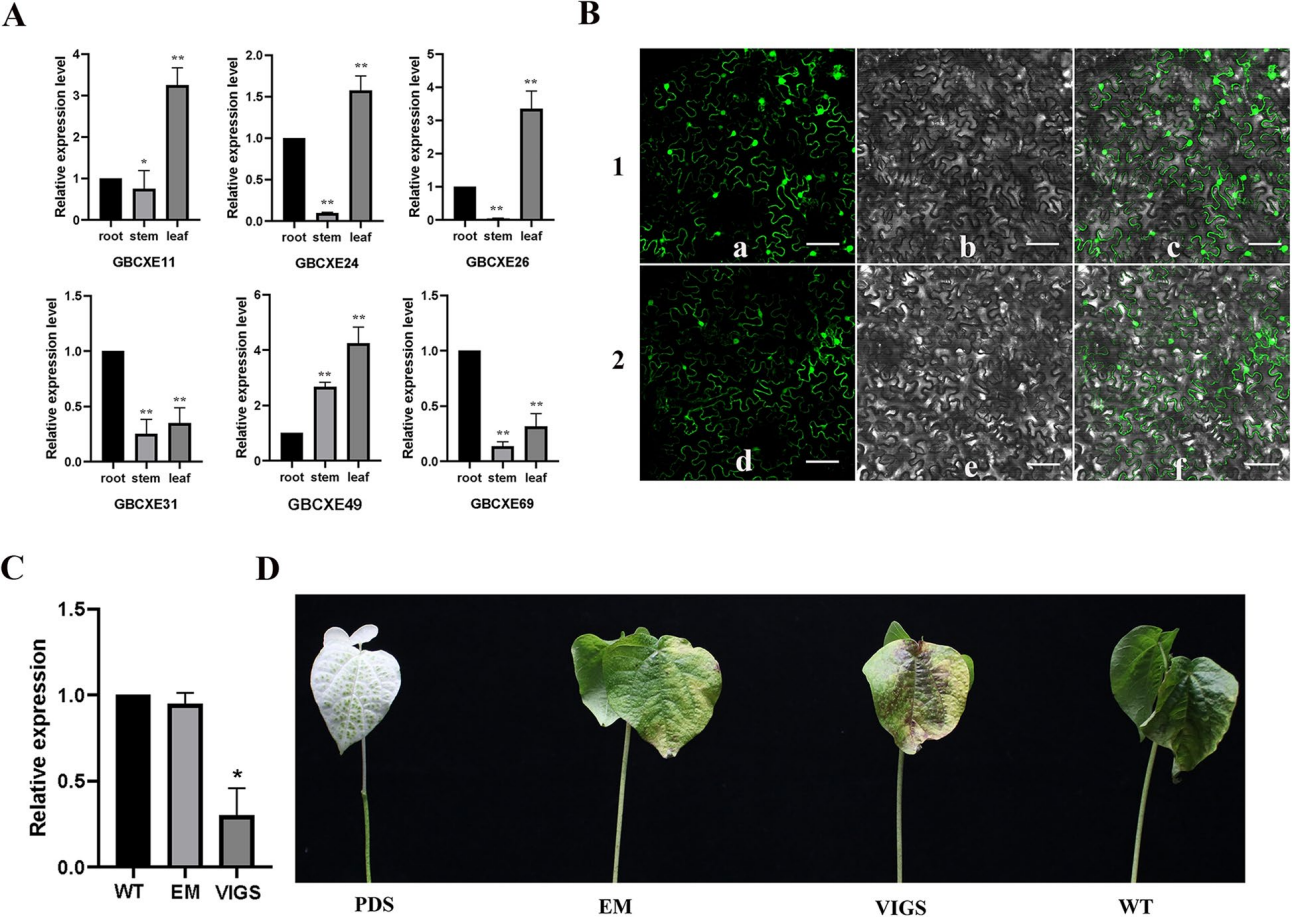
The results of *GBCXE* genes expression analysis and alkaline resistance exploration. **A** The Expression level of *GBCXE* genes in different tissues; **B** Subcellular localization of GBCXE49.1, GFP blank vector(control); 2, *GBCXE49-GFP* a and d were confocal image; b and e were cofocal images under transmitted light; c and f were combined by a-b and d-e, the scale is 50 um; **C** Expression of *GBCXE49* gene in leaves after VIGS infection; **D** The phenotype of cotton under alkaline stress for 2 d after VIGS infection

We randomly selected 6 genes based on different mechanisms in response to salt and alkaline abiotic stress responses and used qRT-PCR to detect their expression patterns in different tissues (roots, stems, leaves). The results showed (Fig. 8A) that *GBCXE31* and *GBCXE69* were expressed at a higher level in roots, and *GBCXE11*, *GBCXE24*, *GBCXE26*, and *GBCXE49* were expressed at a higher level in leaves. It was worth noting that we found that *GBCXE49* had a higher expression level in stems and leaves. This may mean that different *GBCXE* genes played different roles in plant growth and development.

### Analysis of subcellular localization of GBCXE49 protein and VIGS silencing

We combined the differential expression patterns of genes in this family in response to salt-alkali stress and tissue specificity, and selected *GBCXE49* gene with high expression levels in roots and leaves for in-depth study. To determine the expression characteristics of GBCXE49 protein in cells, subcellular localization analysis of the gene was performed, a GFP vector for subcellular localization analysis of GBCXE49 protein was constructed. The experiment was carried out by injecting *GBCXE49-GFP* fusion protein into tobacco leaves. at the same time, GFP empty fusion protein was used as a control, after the leaves were injected for 3 days, the position of the fusion protein was observed by a fluorescent confocal microscope. The result (Fig. 8B) showed that GFP empty and *GBCXE49-GFP* was distributed in the cytoplasm, the result showed that GBCXE49 protein was located at the cytoplasm.

When the cotton cotyledons fully unfolded, the cotton cotyledons were infected with the resuspension of no-load (EM) and VIGE respectively. About 10–15 days after infestation by injection, when true leaves appeared albino (Fig. 8D), the qRT-PCR technology was used to detect *GBCXE49* gene expression in normal plants (WT), EM and VIGS plants, the results showed (Fig. 8C) that the expression level of *GBCXE49* in leaves of WT and EM plants was similar, and was significantly higher than that of VIGS plants in which the *GBCXE49* gene was barely expressed, indicating that the *GBCXE49* gene was mediated silencing. We treated *G. barbadense* WT, EM and VIGS plants with alkaline stress with 50 mM Na_2_CO_3_. At this time, we used uninjected plants (WT) as a control. After treatment for 2 days, we found that all three plants had wilted and the infested VIGS and EM plants were significantly more stressed than WT plants, and the stress was the heaviest in VIGS, followed by EM, and then WT plants. This showed to a certain extent that the silencing of the *GBCXE49* gene affects the tolerance of *G. barbadense* plants to alkaline stress.

## Discussion

Existing studies have found that plant CXE isoenzymes were widely present in different tissues, different organs, different developmental stages and different parts of cells [29, 30]. The research on gene families began in the 1970s, with the purpose of clarifying the relationship between individual or group genetic differences and gene redundancy [31]. The number of *CXE* genes we identified in four cotton species (*G. barbadense*, *G. hirsutum*, *G. arboretum*, *G. raimondii*) were 72, 74, 38, 39, respectively. Here, the number of genes in tetraploid cotton was about the sum of the numbers of two diploids (Fig. S2), which explained the origin of tetraploid cotton to some extent. Through motif and gene structure analysis (Fig. 5), we speculated that the *G. barbadense CXE* gene family was relatively conservative. Researchers found that gene duplication events were caused by polyploidization or tandem and segmented duplication [32]. In our chromosome mapping analysis, we found that the genes of the 6 subgroups were scattered on each chromosome, and there was no *CXE* gene located on chromosome 2 of the diploid subgroup A, which was similar to the tetraploid subgroup A. Interestingly, there was no distribution of the *CXE* gene on chromosome 3 of the diploid D subgroup, but in the two tetraploids, there were *GBCXE43* and *GHCXE44* genes on the chromosome 3 of the D subgroup.

Replication events (tandem duplication, segmental duplication, and whole-genome duplication) played an important role in gene amplification [33, 34]. The subfunction-alization model of repetitive genes believed that after a repetitive event occurs, each repetitive gene not only retains part of the function of the ancestral gene, but also can be divided into complete subfunction and partial subfunction. Complete subfunctionalization meaned that the two repeated genes retain part of the functions of the ancestor genes without overlapping functions, while partial subfunctionalization means that two repeated genes have partial functions overlapping [35]. Sub-functionalized replicating genes generally differentiate at the transcriptional level first, and tend to be subject to strong purification options. As a rule, gene duplication would produce two copies, and under low selective pressure, the gene will evolve, or produce some new functions, which will contribute to the evolution of the species. Replication events were one of the main drivers of evolutionary diversification of genomes and genetic systems [36, 37]. Gene duplications helpd to form new gene functions, which laid the foundation for evolution [38]. The high proportion of repetitive genes in the plant genome just reflects the retention rate of the repetitive modules of the plant species [39]. Exploring the retention mechanism of duplicate genes in plant genomes is still a topic of great interest. Whenever a duplication event occurs, the entire genetic sequence of cotton would double, over time, these redundant genes would be selectively recombined or lost [21]. Part of the repeated genes retained in their offspring could provide the most basic genetic information for the evolution of plants [40]. The calculation of the replication gene Ks showed that as the age of the replication gene increases, the fewer genes have the same cis-acting elements. Our Ka/Ks determination found that about 90% of the *CXE* genes of the four cotton species had a Ka/Ks ratio of less than 1, indicating that the family genes were affected Strong purification options. Combined with the results of collinearity analysis (Fig. 3), we identified a total of 238 pairs of repeats in the four cotton species. among the 20 *Arabidopsis CXE* genes, 4 were generated by tandem repeats, and the genes generated by tandem repeats accounted for 20% of the entire *CXE* family [2], combined with our results and *Arabidopsis*, fragment duplication was the main reason for gene amplification of *GBCXEs*.

Plant *CXEs* are involved in the activation of herbicide active substances, the metabolism of plant hormone signal substances and biological stress and other processes to play important biological functions. Early research found that plant *CXE* can demethylate inactive methyl salicylate and methyl jasmonate to form active salicylic acid and jasmonic acid [27, 41]. In addition, a tomato-derived esterase was found to be able to cleave the methyl jasmonate signal molecule [42], other studies have also confirmed that carboxylesterase was involved in the hydrolysis of tomato volatile esters and acyl sugars in tomato glandular hair [43, 44]. Later related studies showed that the gene *VvMJE1* with methyl jasmonate (MeJA) esterase activity was cloned in *Vitis vinifera*, which was significantly up-regulated after cold treatment and UV-B treatment [28]. In our research, there were a lot of light, low temperature, drought, defense stress response, hormone response elements in the *GBCXE* gene promoter (MeJA, salicylic acid, ABA, IAA, GA). We speculated that *CXE* family genes may be related to abiotic stress and hormone regulation pathways. In *Zea mays*, CXE protein can also regulate the metabolism of gibberellin (GA20) glycosyl [45]. Kowalczyk found that *CXE* regulates IAA metabolism in the immature endosperm tissue of *Zea mays* [46]. Previous studies have proposed that indole acetic acid (IAA) in plants is stored in the form of sugar ester methyl esters and other forms, and *CXE* can act on sugar ester methyl esters to generate active auxins to maintain the balance of plant growth and development [47]. *GA2ox* was an oxidase with regulatory function in the third stage of gibberellin synthesis [48]. In our protein interaction network (Fig. 7), GA2ox protein and carboxylesterase AtCXE protein also interact with each other. In our RNA-seq data, the up- and down-regulation trends of most *GBCXE* family genes in response to the two stresses are basically the same, but the degree of response was different, and the expression levels were different. We speculated that this result was caused by the difference in pH between the two sodium salts. This also confirmed the difference in the response of *G. barbadense* to the two stresses. The analysis of our promoter action elements combined with the research obtained further shows that carboxyl esterase may also play a role in plant signal transduction pathways.

*CXE* had a wide range of substrate catalytic activity, a wide range of functions, and participates in a variety of different biological processes [49, 50], and *CXE* with different functions would have different positioning in plants, moreover, the expression and role of different *CXE* genes in different tissues of plants were not the same. The *GBCXEs* we selected, *GBCXE31*, *GBCXE69* were highly expressed in roots, *GBCXE11*, *GBCXE24*, *GBCXE26* were highly expressed in leaves, and *GBCXE49* was highly expressed in stems and leaves, combined with the evolutionary relationship between species, it was not difficult to find that the *GBCXE49* gene and the *CXE6*, *CXE17* genes of *Arabidopsis* were located in the same group. In addition, the subcellular localization results showed that *GBCXE49* was localized in the cytoplasm. We learned that the *PpCXE1* gene in peaches, and its subcellular location indicates that it was also present in the cytoplasm [51]. *Arabidopsis* contained a variety of carboxylesterases with biological activity of foreign organisms (herbicide esters), and it has been verified by tDNA knock-out that the role of *AtCXE12* on the bioactivation of herbicides in *Arabidopsis* was confirmed [52]. Another study showed that the carboxylesterase (CXE) in the seedling *Cucurbita moschata* was involved in the metabolism of di-n-butyl phthalate (DnBP) [53]. We combined transcriptome analysis to select the *GBCXE49* gene that actively responds to alkaline stress and is highly expressed in roots and leaves, through the VIGS technology to silence it in cotton, it was finally discovered that the gene may be involved in the regulation mechanism of cotton alkaline stress.

## Conclusions

In summary, we have identified the *CXE* family genes in four cotton species and analyzed their evolutionary relationship. The gene structure, chromosome distribution, phylogenetic relationship, cis-acting elements, and collinearity relationship of *G. barbadense GBCXEs* were compared, which greatly enriched our understanding of the cotton *CXE* gene family. Through the analysis of the cis-acting elements in the promoter of *GBCXE* genes, it was speculated that *GBCXE* genes may be involved in stress defense response, abiotic stress and plant hormone signal transduction mechanism. Combined with transcriptome analysis, we selected 6 *GBCXE* genes for simple tissue-specific analysis, and VIGS silenced the *GBCXE49* gene that significantly responded to alkaline stress, and finally determined that *GBCXE49* gene may be involved in the alkaline stress response of *G. barbadense*, which provided a basis for the study of the molecular mechanism of *CXE* genes in abiotic stress in plants.

## Materials and methods

### Identification of G. *barbadenseCXE* gene members

We download the genome annotation file of *G. barbadense* from Cotton FGD (Cotton Functional Genomics Database) (http://www.cottonfgd.org/). The Hidden Markov Model (HMM) (version 3.0) configuration file of PF07859 was downloaded from Pfam (https://pfam.xfam.org/). Then, we used the HMMER 3.0 software with default parameters and settings to obtain the *CXE* gene and protein sequence of Pfam PF07859. This type of gene was likely to belong to the *CXE* gene family. We used the online websites Pfam (https://pfam.xfam.org/) and SMART (http://smart.emblheidelberg.de/) to analyze the gene structure, then conduct a comprehensive analysis, and further screen to determine the gene family member. Online tools, such as CELLO *ver*.2.5 (http://cello.life.nctu.edu.tw/) [54], TargetP (http://www.cbs.dtu.dk/services/TargetP/), WOLF-PSORT (https://wolfpsort.hgc.jp/)were used to predict the subcellular location information of *GBCXEs*. We also searched for other features of the *G. barbadense CXE* gene by using the Cotton Functional Genome Database (CottonFGD). The online website ExPASy-ProtParam (https://web.expasy.org/protparam/) was used to analyze Isoelectric point (pIs), Molecular weight (MWs), exon/intron length, mean hydrophilicity and charge.

### Gene system evolution analysis

To study the evolutionary relationship between *CXE* genes of different cotton species, similarly, we used the bio-analysis software HMMER 3.0 to obtain the homologous genes of *Gossypium hirsutum*, *Gossypium arboreum*, and *Gossypium raimondii*. The *Arabidopsis* genome data was obtained from the database *TAIR* (https://www.arabidopsis.org/), and the *AtCXE* gene protein sequence was obtained using the Hidden Markov Model Abhydrolase_3 of PF07859 as the screening condition. We used the online tool CD-Search (https://www.ncbi.nlm.nih.gov/Structure/bwrpsb/bwrpsb.cgi) to screen and obtain *AtCXE* gene members, all the protein sequences were further analyzed in the SMART (http://smart.embl-heidelberg.de/). The protein sequence was named according to its position on the chromosome [55], aligned by using MEGA7.0 software, and the rootless phylogenetic tree was also constructed by MEGA7.0. The parameters were set as follows: neighbor-joining statistics method, bootstrap replication 500, *GBCXEs* intraspecific and interspecific phylogenetic relationship have been constructed in the same way.

### Analysis of protein conserved domains and gene structure

For the *CXE* family genes of *G. barbadense*, we used the MEME (http://meme-suite.org/) website to identify the conserved sequence of the protein. The maximum motif parameter of the gene was 10, the rest of the parameters remain unchanged, and the domain files of the *GBCXE* family genes were obtained accordingly. The domain MAST file, the gene evolution relationship file, and the *G. barbadense* genome gff3 file downloaded from the MEME website were used to draw the protein domain picture through the TBtools tool.

### Chromosome location

*G. barbadense* reference genome gff3 file and gene ID files were used to map the chromosome positions of family members through TBtools software, the position of *CXEs* in the chromosomes of *G. hirsutum*, *G. arboreum* and *G. raimondii* was successfully mapped by the same method. The genome sequences of *G. hirsutum*, *G. arboreum*, and *G. raimondii* are all downloaded from Cotton FGD (http://www.cottonfgd.org/).

### Analysis of the promoter region and differentially expressed genes of *GBCXEs*

We used the reference genome and the genome sequence file, and obtained the 2000 bp DNA sequence upstream of the *GBCXEs* using the software Tbtools. The Plant CARE website (http://bioinformatics.psb.ugent.be/webtools/plantcare/html/) was used to predict the cis-regulatory elements in the promoter region of the gene *GBCXEs.* Metabolism, plant growth and development related cis-acting elements were screened and analyzed. Similarly, the software TBtools was used for mapping [56].

According to RNA-seq data, the differentially expressed genes of *G. barbadense* under salt (NaCl, 100 mM) and alkaline (Na_2_CO_3_, 50 mM) stress were analyzed. The genes that *GBCXE* family members respond to stress were screened out, and heat maps, phylogenetic trees and cis-acting elements were generated using TBtool software by using the number of fragments per kilobase exon (FPKM). The seeds of *G. barbadense* alkali-resistant material (Jiza 67) were provided by the Crop Genetics and Breeding Laboratory of Xinjiang Agricultural University, according to the RN38 EASYspin Plus plant RNA rapid extraction kit instructions of Beijing Adelaide Biotechnology, the total RNA of the root, stem, and leaf tissues were extracted separately, according to HiScript® III RT SuperMix for qPCR (+ gDNA wiper) instructions for preparing cDNA. The equipment used for qRT-PCR was Applied Biosystems@ 7500 Fast, and the fluorescence quantification kit was ChamQ Universal SYBR qPCR Master Mix. Primers were listed in supplementary Table S2. The ΔΔCt method [57] was used to calculate the processing results.

### Collinearity analysis of *CXEs* in four cotton species

We the gff file of the target genome was prepared and merged with the TBtool software, then the protein sequence alignment of the genome and then the gene pair files of each two species were obtained (Ga-Ga, Ga-GH, Ga-Gr, Ga-GB, GB-GH, GB-Gr, GB-GB, Gr-GH, Gr-Gr, GH-GH). The TBtool software was used to analyze the collinearity of the repetitive gene pairs of 4 cotton species (*G. barbadense*, *G. hirsutum*, *G. arboreum*, *G. raimondii*). The chromosome length file and the genome alignment filewere used for finally the collinearity result visualization.

### Subcellular localization of *GBCXE* gene and VIGS silencing

The *GBCXE49*-GFP vector was constructed by primers (supplementary Table S2). When Nicotiana tabacum was cultured for 3–4 weeks, we took an appropriate amount of activated agrobacterium containing the target gene-containing subcellular targeting vector and added it to the resistant LB culture (Kanamycin 50 mg/L and Rifampicin 25 mg/L) liquid medium, cultivate to OD 600 to about 0.8–1.0, centrifuge to collect the bacterial liquid and resuspend the bacterial cell with the resuspension liquid, and inject it into the leaves. The infected plants were cultured for 24 h in the dark, and then cultured normally for 2 days. The laser confocal was used to observe the green fluorescence of GFP near the injection site of the leaves.

Using the cloning vector *t-GBCXE49* as a template, the primer *inGBCXE49-V* (supplementary Table S2) was used for amplification to obtain VIGS fragments. The VIGS of *GBCXE49* was silenced by in-FuSion Insert the fragment into the linear PYL156 vector. After planting Jiza 67 until two cotyledons were flattened, a disposable medical syringe was used to inject VIGS on the back of the leaves (injection area was more than 80%), and then incubated in the dark at 28 °C for 24 h. Ensure the normal growth of seedlings at a temperature (28 14 h/25 10 h).

## Supplementary Information

**Additional file 1:** **Figure S1.** Distribution of *CXE* genes in four cotton species. **Figure S2.** A breakdown of the number of *CXE* genes among species. **Table S1.** Attached table of physical and chemical properties. **Table S2.** Primer sequence. **Table S3.** Tandem repeats and fragment repeats in four cotton species. **Table S4.** Gene pairs of ten combinatorial. **Table S5.** Prediction of duplicated gene pairs involved in different combinations from four *Gossypium* species. **Table S6.** Statistics of promoter cis-element. **Table S7.** AlkVSCK_Gene_differential_expression. **Table S8.** SalVSCK_Gene_differential_expression.

## Data Availability

The genomic data of cotton, *Arabidopsis thaliana* in the article can be downloaded from Cotton FGD (https://cottonfgd.org/) and TAIR (https://www.arabidopsis.org/) respectively. The analysis software, analysis methods and datasets generated are available from the corresponding author on reasonable request. The RNA seq data of Jiza 67 under salt (NaCl) and alkaline (Na_2_CO_3_) treatment had been deposited in the database of the National Center for Biotechnology Information (NCBI) ( https://dataview.ncbi.nlm.nih.gov/object/PRJNA734700?reviewer=7s2hkmdclmc9u9g76hevgpml5b) under accession number PRJNA734700. The databases were closed, I had received administrative permission to access and use these. All the databases will be released at 2024–07-06.

## Abbreviations

CXE: Carboxylesterase
GA: Gibberellin
IAA: Auxin
VIGS: Virus induced gene silencing
PDS: Phytoene desaturase
EM: Empty vector
WT: Wild type
At: *Arabidopsis thaliana*
GB: *Gossypium barbadense*
GH: *Gossypium hirsutum*
Ga: *Gossypium arboreum*
Gr: *Gossypium raimondii*
MEME: Multiple Em for Motif Elicitation
MeJA: Methyl Jasmonate
FPKM: Fragments Per Kilobase of exon model per Million mapped fragments
