# Supplementary material for: Genome-wide expression analysis of carboxylesterase (CXE) gene family implies GBCXE49 functional responding to alkaline stress in cotton
Source: BMC Plant Biol. 2022 Apr 12;22:194. doi: 10.1186/s12870-022-03579-9 (PMC9004025; doi:10.1186/s12870-022-03579-9)
Supplement: Supplementary file 1 — Additional file 1: Figure S1. Distribution of CXE genes in four cotton species. Figure S2. A breakdown of the number of CXE genes among species. Table S1. Attached table of physical and chemical properties. Table S2. Primer sequence. Table S3. Tandem repeats and fragment repeats in four cotton species. Table S4. Gene pairs of ten combinatorial. Table S5. Prediction of duplicated gene pairs involved in different combinations from four Gossypium species. Table S6. Statistics of promoter cis-element. Table S7. AlkVSCK_Gene_differential_expression. Table S8. SalVSCK_Gene_differential_expression. [file 12870_2022_3579_MOESM1_ESM.zip › Summary of Figures and Tables.docx]

**Summary of Figures and Tables**

**Fig. 1.** Phylogenetic tree of *CXE* family members.

**Fig. 2.** Analysis of phylogenetic tree, conserved domains and gene structure of *GBCXEs.*

**Fig. 3.** Analysis of promoters and differentially expressed genes of *GBCXEs* family.

**Fig. 4.** Chromosomal location of *CXE* genes in four cotton species.

**Fig. 5.** The collinearity of *CXE* genes within and among the four cotton species genomes.

**Fig. 6.** Select the radar chart for pressure (Ka/Ks) analysis.

**Fig. 7.** Interaction network of GBCXE proteins.

**Fig. 8.** The results of *GBCXE* genes expression analysis and alkaline resistance exploration.

**Supplementary figure and table**

**Fig.S1** Distribution of *CXE* genes in four cotton species

**Fig.S2** A breakdown of the number of *CXE* genes among species

**Table S1. Attached table of physical and chemical properties**

**Tab. S2** Primer sequence

**Tab. S3** Tandem repeats and fragment repeats in four cotton species

**Tab. S4** Gene pairs of ten combinatorial

**Tab. S5** Prediction of duplicated gene pairs involved in different combinations from four *Gossypium* species.
