# Supplementary material for: Genome-wide expression analysis of carboxylesterase (CXE) gene family implies GBCXE49 functional responding to alkaline stress in cotton
Source: BMC Plant Biol. 2022 Apr 12;22:194. doi: 10.1186/s12870-022-03579-9 (PMC9004025; doi:10.1186/s12870-022-03579-9)
Supplement: Supplementary file 1 — Additional file 1: Figure S1. Distribution of CXE genes in four cotton species. Figure S2. A breakdown of the number of CXE genes among species. Table S1. Attached table of physical and chemical properties. Table S2. Primer sequence. Table S3. Tandem repeats and fragment repeats in four cotton species. Table S4. Gene pairs of ten combinatorial. Table S5. Prediction of duplicated gene pairs involved in different combinations from four Gossypium species. Table S6. Statistics of promoter cis-element. Table S7. AlkVSCK_Gene_differential_expression. Table S8. SalVSCK_Gene_differential_expression. [file 12870_2022_3579_MOESM1_ESM.zip › Supplementary figure and table.docx]

**Supplementary figure and table**


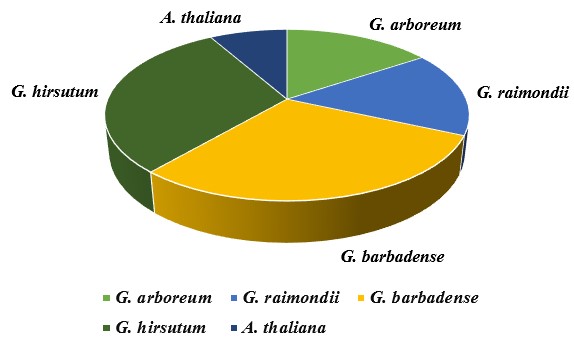


**Fig.S1** Distribution of CXE genes in four cotton species


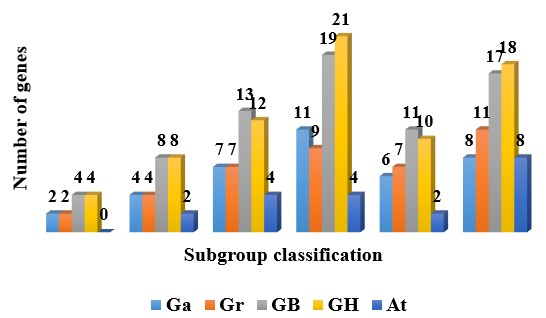


**Fig.S2** A breakdown of the number of CXE genes among species

**Tab. S2** Primer sequence

| Gene | Primers for PCR (5'-3') | |
| --- | --- | --- |
| GBCXE11 | | F: GTAGGAGCTGAGGCAGAAGT |
|  |  | R: ATCCTTGGCAAGAGCTTCCT |
| GBCXE26 | | F: CCTCAACGAGTGCAAGATGG |
|  |  | R: GGACTCTGCGGTCAAGAAAC |
| GBCXE31 | | F: AGCCTACCAGCTTCGGATAC |
|  |  | R: CCCAAGGCAGCACAATACTC |
| GBCXE49 | | F: AGGTGATAGCTCAGGAGGGA |
|  |  | R: AACGAACCCTGGGTGGATAG |
| GBCXE24 | | F: GCGGGAGTTTCACTCATTCC |
|  |  | R: TCGGCGATAGTTCACTGACA |
| GBCXE69 | | F: TTGGAGTTGCTACCACGAGT |
|  |  | R: CCCATCTTCGTAAGCAGCAG |
| UBQ7 | | F: GACCTACACCAAGCCCAAGAAG |
|  |  | R: TGAGCCCACACTTACCACAATAGT |
| GBCXE49-GFP | | F: AGAACACGGGGGACTCTAGAATGGTTCAAGAAAAGAAGC |
|  |  | R: TCTTCTCCTTTACCCATGTTAATTAAATGCTTATTGATGAACAC |
| InGBCXE49-V | | F: TGAGTAAGGTTACCGAATTCATGATGATGGCTCAGTCG |
|  |  | R: AATGTCTTCGGGACATGCCCGGGGGTAAACCGAGACGACAAT |

**Tab. S3** Tandem repeats and fragment repeats in four cotton species

| combination | fragment repeats | tandem repeats |
| --- | --- | --- |
| Ga-Ga | 22 | 3 |
| Gr-Gr | 7 | 5 |
| GB-GB | 124 | 7 |
| GH-GH | 83 | 7 |

**Tab. S4** Gene pairs of ten combinatorial

| combination | gene pairs |
| --- | --- |
| Ga-GH | 102 |
| Ga-Gr | 53 |
| Ga-GB | 154 |
| GB-GH | 286 |
| GB-Gr | 159 |
| Gr-GH | 142 |
| Ga-Ga | 25 |
| Gr-Gr | 12 |
| GB-GB | 131 |
| GH-GH | 90 |

**Tab. S5** Prediction of duplicated gene pairs involved in different combinations from four *Gossypium* species.

| Pairs | Positive | Pure Selection | Total |
| --- | --- | --- | --- |
| Ga-Ga | 0 | 23 | 23 |
| Ga-GH | 3 | 88 | 91 |
| Ga-Gr | 1 | 47 | 48 |
| Ga-GB | 3 | 122 | 125 |
| GB-GH | 6 | 223 | 229 |
| GB-Gr | 3 | 121 | 124 |
| GB-GB | 2 | 96 | 98 |
| Gr-GH | 2 | 119 | 121 |
| Gr-Gr | 0 | 11 | 11 |
| GH-GH | 1 | 77 | 78 |
| Total | 21 | 927 | 948 |
| percentage | 2.22 | 97.78 | 100 |
